# Impact of COVID-19 pandemic measures on hospitalizations and epidemiological patterns of twelve respiratory pathogens in children with acute respiratory infections in southern China

**DOI:** 10.1186/s12879-025-10463-y

**Published:** 2025-01-22

**Authors:** Wei Wang, Xiaojuan Luo, Zhenmin Ren, Xiaoying Fu, Yunsheng Chen, WenJian Wang, Yanmin Bao, Yuejie Zheng, Ke Cao, Jiehua Chen

**Affiliations:** 1 Department of Respiratory, Shenzhen Children′s Hospital, Shenzhen, 518038 China; 2 Department of Clinical Laboratory, Shenzhen Children′s Hospital, Shenzhen, 518038 China

**Keywords:** COVID-19, Non-pharmaceutical interventions, Pathogens, Epidemiology, Children, Acute respiratory infection

## Abstract

**Objectives:**

To investigate the impact of COVID-19 pandemic measures on hospitalizations and the alterations and persistence of the epidemiological patterns of 12 common respiratory pathogens in children during the COVID-19 pandemic and after the cessation of the “zero-COVID-19” policy in southern China.

**Methods:**

Respiratory specimens were collected from hospitalized children with acute respiratory infections at Shenzhen Children’s Hospital from January 2020 to June 2024. Twelve common respiratory pathogens were detected using multiplex PCR. Data on demographic characteristics, pathogen detection rates, epidemiological patterns, co-infections, and ICU admission rates were compared between the ‘during COVID-19’ period (Phase 1: January 2020 to December 2022) and the ‘post COVID-19’ period (Phase 2: January 2023 to June 2024).

**Results:**

In Phase 2, there was a significant increase in average annual cases, with a higher median age of affected children, higher pathogen detection rates, and increased co-infection rates compared to Phase 1. The epidemiological patterns of most pathogens were altered by the COVID-19 pandemic. Human Parainfluenza Virus, Human Metapneumovirus, Human Bocavirus (HBOV), and Human Coronavirus remained active during Phase 1, while *Mycoplasma pneumoniae* (*Mp*) and Adenovirus (ADV) were low, and Respiratory Syncytial Virus (RSV) lacked a seasonal peak in 2022. In Phase 2, *Mp*, ADV, and RSV experienced outbreaks, with *Mp*’s high prevalence continuing into 2024. RSV showed out-of-season epidemics for two consecutive years. Influenza A (H1N1), Influenza A (H3N2), and InfB lost their seasonal patterns during Phase 1 but reemerged and regained their seasonal characteristics in 2023–2024. ICU admission rates did not significantly differ between the two phases, except for HBOV, which had higher rates in Phase 2.

**Conclusion:**

The epidemiological patterns of various respiratory pathogens were affected by the COVID-19 pandemic to varying degrees. Pathogens suppressed during the pandemic experienced outbreaks or out-of-season epidemics after the lifting of non-pharmaceutical interventions, with *Mp* and RSV continuing into the second year and HBOV associated ICU admission rates increasing in the post-pandemic era. Continuous monitoring of these patterns is essential to understand the duration of these effects and to inform effective response strategies.

## Background

Respiratory infections are major cause of pediatric hospitalization and pose a significant threat to global child health. Respiratory viruses and *Mycoplasma pneumoniae* (*Mp*) are particularly prevalent in children with community-acquired pneumonia (CAP), frequently leading to severe illness and, in some cases, mortality [1]. The COVID-19 pandemic has presented unprecedented challenges to global public health systems and has concurrently reshaped the epidemiological patterns of other respiratory pathogens [2].

During the pandemic, a suite of non-pharmaceutical interventions (NPI) measures were implemented in China and globally to mitigate the spread of SARS-CoV-2. These strategies, including mask-wearing, hand hygiene, social distancing, and lockdowns, profoundly influenced not only the trajectory of COVID-19 but also, serendipitously, the prevalence of other respiratory pathogens [3–5]. This broad-spectrum reduction in pathogen transmission has been associated with the emergence of “immunity debt,” a concept describing the diminished protective immunity due to prolonged reduced exposure to pathogens, thereby increasing the vulnerability of the population to a range of diseases [6]. The relaxation of NPI measures has been correlated with observed resurgences and the occurrence of out-of-season or unusually severe respiratory infections, as documented in recent literature [4, 7–9]. The altered epidemiological patterns of multiple respiratory pathogens in the post-pandemic era and their ongoing impact have garnered significant attention, underscoring the importance of sustained surveillance.

Shenzhen, situated in the southern part of China, has a population exceeding seventeen million. The Shenzhen Children’s Hospital is the region’s only pediatric medical facility. Since late 2019, multiplex polymerase chain reaction (PCR) testing for 12 respiratory pathogens has been conducted on all children admitted with acute respiratory infections (ARI), yielding a robust dataset encompassing these pathogens. Previous research conducted by our department by January 2022 has documented that the stringent lockdown measures instituted during the COVID-19 pandemic had a profound impact on the epidemiology of respiratory pathogens, with a notable decrease in the incidence of *MP*, influenza A, and adenovirus (ADV) infections. Conversely, infections with respiratory syncytial virus (RSV) and human parainfluenza virus (HPIV) increased [10, 11]. The COVID-19 pandemic lasted until the end of 2022, when the “zero-COVID-19” policy ended and restrictions were fully lifted as of January 2023. This study aims to delineate the epidemiological profiles of these 12 respiratory pathogens among hospitalized children with ARI during the COVID-19 pandemic (2020–2022), the first year after the lifting of COVID-19 restrictions (2023), and the second year from Jan to Jun (2024). Insights into the fluctuations in respiratory pathogen prevalence will contribute to the formulation of subsequent public health initiatives and prophylactic strategies.

## Methods

### Study subjects and ethics statement

**Fig. 1:**
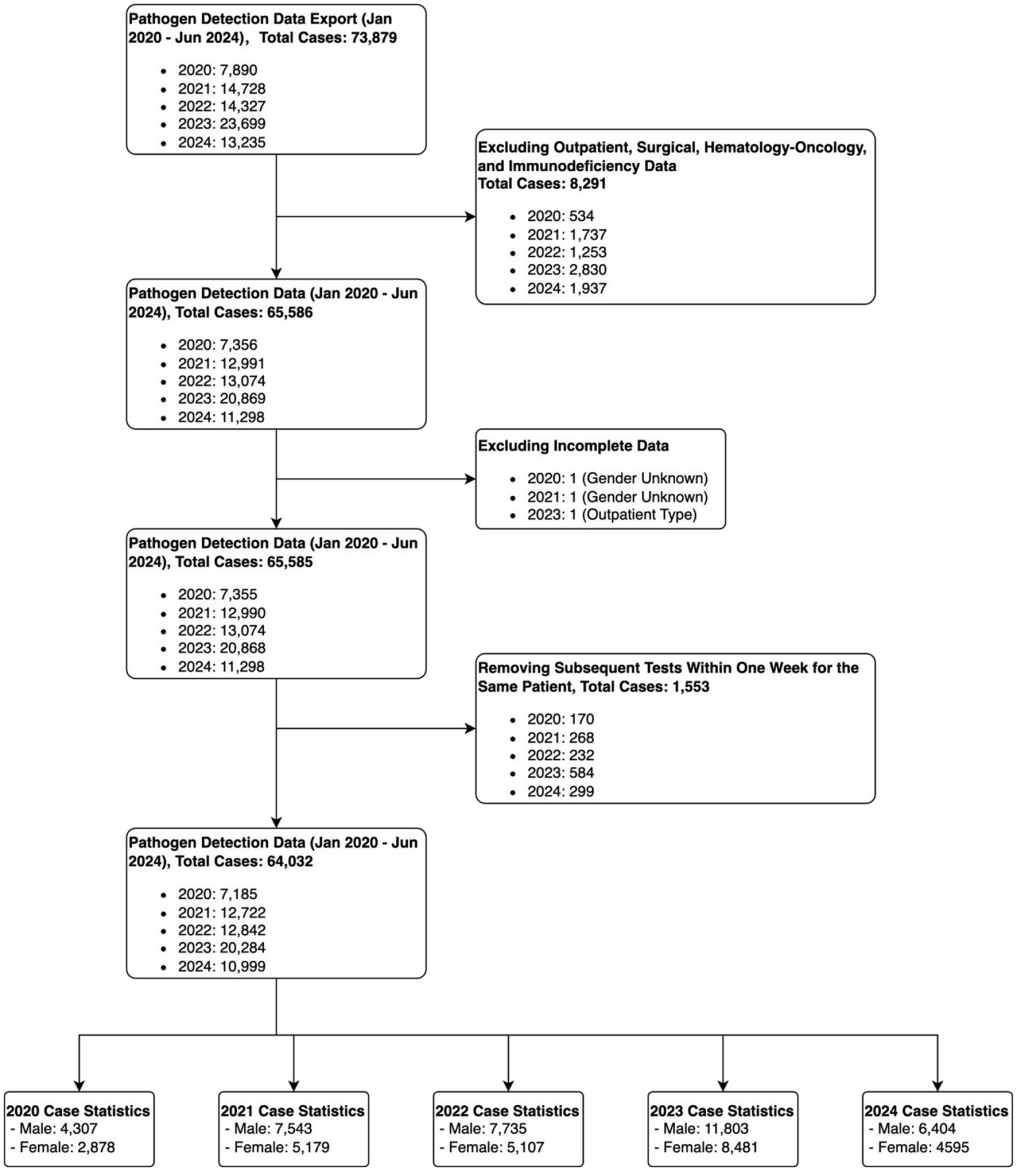
The flowchart of case enrollment

This study enrolled patients diagnosed with ARI and admitted to the pediatric wards of Shenzhen Children’s Hospital from January 2020 to June 2024. Inclusion criteria encompassed individuals presenting with one or more respiratory symptoms affecting the respiratory tract, including rhinorrhea, sore throat, cough, wheezing, dyspnea, tachypnea, with or without fever. Exclusion criteria were applied as follows: (1) hematological malignancy, or a history of hematopoietic stem cell transplantation; (2) repeat testing for the same pathogen within a one-week period; and (3) incomplete age or gender information (Fig. 1). Laboratory and demographic data for the enrolled patients were extracted from electronic medical records. The study protocol was reviewed and granted approval with a waiver of informed consent by the Shenzhen Children’s Hospital Ethical Committee. The research was conducted in compliance with pertinent ethical guidelines and regulatory standards.

The patients were divided into six age groups: (1) newborn(< 1 month), (2) infants (≥ 1 month, < 1 year), (3) toddlers (≥ 1 year, < 3 years), (4) pre-school children (≥ 3 years, < 6 years), (5) school-aged children (≥ 6 years, <14years) and 6)adolescents (≥ 14 years, < 18years).

### Specimen detection

A SureX^®^ 13 Respiratory Pathogen Multiplex Kit (Health Gene Technologies, Ningbo, China) was used to detect respiratory pathogens according to the recommended protocol, including RSV, ADV, HPIV, general influenza virus A (InfA), InfA(H1N1), InfA(H3N2), influenza B (InfB), human metapneumovirus (HMPV), human rhinovirus (HRV), human bocavirus (HBOV), human coronavirus (HCOV, including strains 229E, OC43, NL63, and HKU1), *MP*, and *Chlamydia* (*Ch*).

### Statistical analyses

The “zero-COVID-19” policy was implemented at the beginning of 2020 and ended at the beginning of 2023 in China, thus the study periods were categorized as “During COVID-19” period (from January 2020 to December 2022) and “Post COVID-19” period (from January 2023 to June 2024). The detection rates and co-infection rates of these 12 prevalent respiratory pathogens were compared between these two periods. The epidemiological patterns of these pathogens were examined through the monthly distribution of detection rates. The differences in detection rates of these pathogens by gender and age, as well as the Intensive Care Unit (ICU) admission rates, were compared between these two phases.

Statistical analyses were conducted using SPSS 22.0 software (IBM, New York, United States). Categorical variables were expressed as numbers (%), while continuous variables were expressed as medians (interquartile range). Proportions for categorical variables (detection rates of pathogens, sex, and age) were compared using the chi-square test. Median ages were compared using the Mann-Whitney U test. All tests were two-tailed, and a value of *P* < 0.05 was considered statistically significant.

## Results

### Patient characteristics and detection rates during and post the COVID-19 periods

**Fig. 2:**
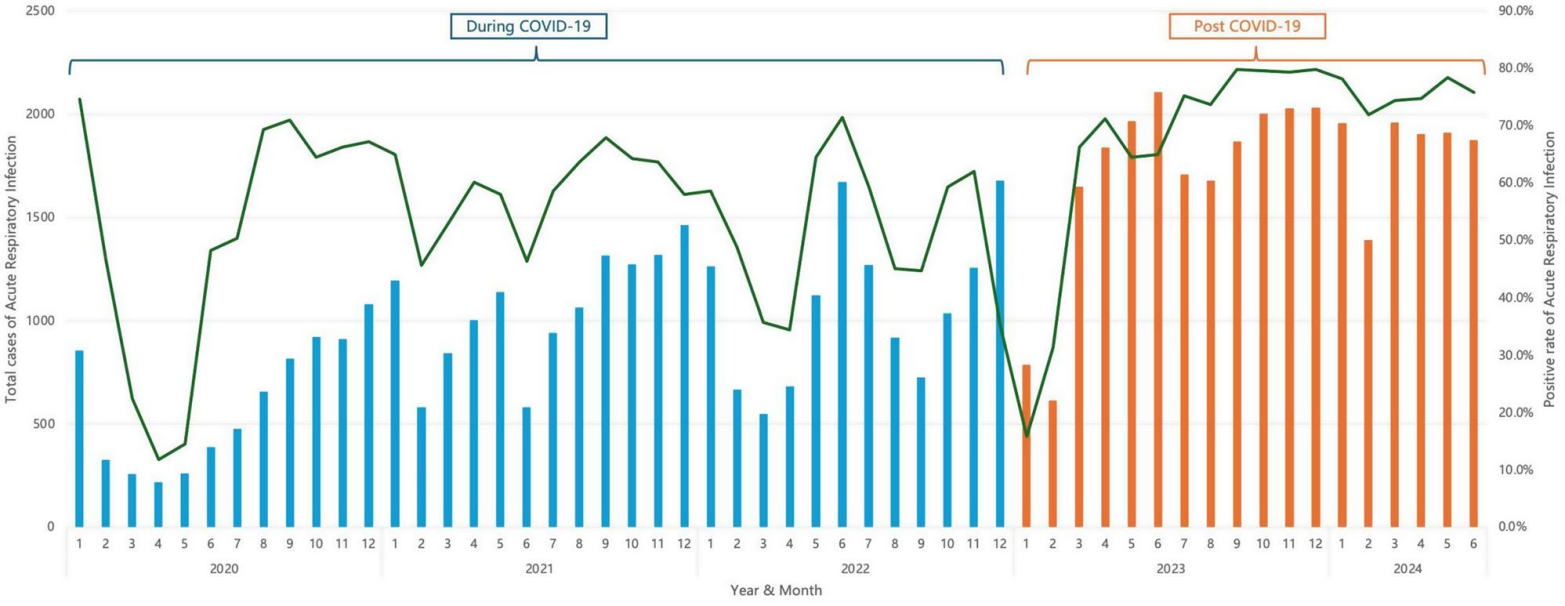
ARI cases (column) and Positive rate(line) of ARI monthly from 2020 to 2024(from Jan to Jun)

A total of 64,032 children with ARI were included in this study, with 32,749 cases from Jan 2020 to Dec 2022 (during COVID-19), and 31,283 cases from Jan 2023 to Jun 2024 (post COVID-19). The proportion of females and the median age increased in the post COVID-19 period. This trend culminated in a 91% increase in ARI hospital admissions yearly in the post COVID-19 period when compared to that during COVID-19. The pathogen detection rates and co-infection rates were significantly higher in the post COVID-19 period than during COVID-19. There was no difference in ICU admission rates between the COVID-19 and post COVID-19 periods (Table 1). Except for January and February 2023, when there was a nationwide surge in SARS-CoV-2 infections following the lifting of restrictions, there was a substantial rise in monthly ARI and pathogen detection rates throughout 2023 and into 2024 (Fig. 2).

**Table 1:** The clinical characteristics and pathogen detection rates during and post the COVID-19 periods

	During COVID-19				Post COVID-19			χ²/U	*P*
	2020	2021	2022	Overall	2023	2024(from Jan to Jun)	Overall		
ARI cases	7185	12,722	12,842	32,749	20,284	10,999	31,283		
Positive cases	4306	7651	6893	18,850	14,199	8333	22,532		
Positive rate	59.9%	60.1%	53.7%	57.6%	70.0%	75.8%	72.0%	1464.875	< 0.001
Co-infection cases	542	801	707	2050	2828	1910	4738		
Co-infection rate	7.5%	6.3%	5.5%	6.3%	13.9%	17.4%	15.1%	771.488	< 0.001
Gender									
Male	4307	7543	7735	19,585(59.9%)	11,803	6404	18,207(58.1%)		
Female	2878	5179	5107	13,164(40.1%)	8481	4595	13,076(41.9%)		
Male: Female				1:0.67			1:0.72	16.986	< 0.001
Age	1.75(0.67, 3.75)	2.17(0.75, 4.25)	2.58(0.90, 4.83)	2.17(0.75, 4.33)	3.67(1.25, 6.67)	3.50(1.00, 6.42)	3.67(1.17, 6.58)	407,937,370	<0.0001
ICU case	330	502	537	1369	799	425	1224		
ICU admission rate	4.6%	3.9%	4.2%	4.2%	3.9%	3.9%	3.9%	2.949	0.086

### Prevalence of respiratory pathogens during and post the COVID-19 periods

**Fig. 3:**
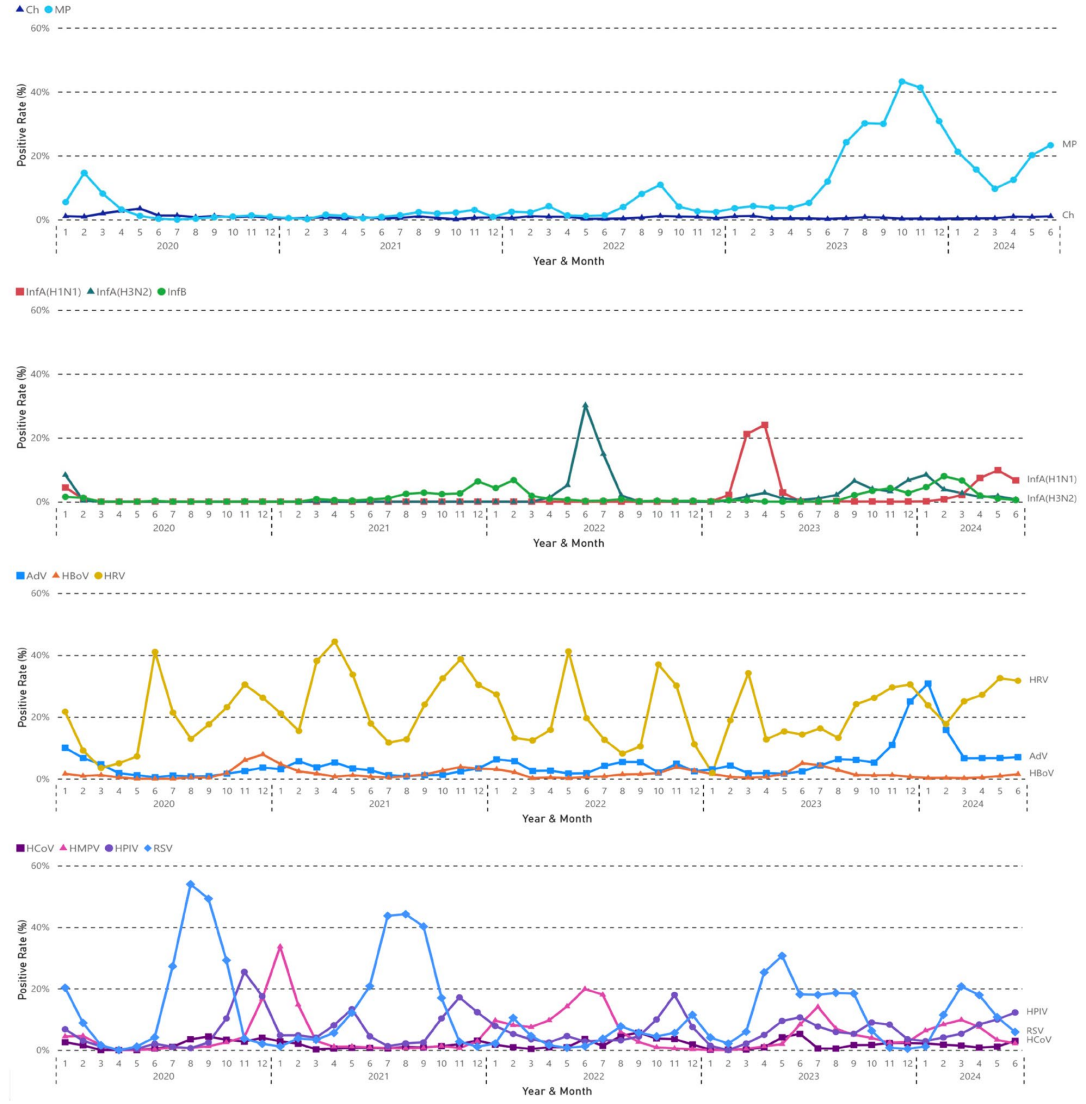
The epidemiological pattern of each respiratory pathogen monthly from 2020 to 2024(from Jan to Jun)

Figure 3 shows the epidemiological pattern of each pathogen. Except for *Ch*, which maintained a consistently low prevalence, all other pathogens experienced an increase in detection rates within a few months after the lifting of restrictions in 2023.

The prevalence of *MP* significantly decreased during COVID-19. However, a subsequent outbreak was observed after the lifting of restrictions in 2023, persisting into 2024. The detection rate of *Ch* remained consistently low throughout the study period.

All influenza viruses disappeared in 2020 during COVID-19 until the winter of 2021, when InfB re-emerged. An outbreak of InfA (H3N2) occurred from May to July 2022. An outbreak of InfA (H1N1) occurred from February to April 2023, soon after the lifting of restrictions. InfA (H1N1), InfA (H3N2), and InfB all re-emerged in winter 2023 and spring 2024.

HRV showed a regular and consistent prevalence pattern during COVID-19, with biannual peaks in April-June and October-November. An earlier peak was observed in March 2023, soon after the lifting of restrictions, and the detection rate remained high through 2024. HBOV also exhibited regular seasonal peaks, typically in November to December, with an atypical peak noted in June 2023 following the lifting of restrictions, but not in June 2024. The detection rates of ADV were low during COVID-19, increased since July 2023, and then a notable outbreak occurred in winter 2023.

RSV outbreaks occurred seasonally with a peak in August to September 2020 and July to September 2021, which was absent in 2022. An out-of-season RSV outbreak emerged in February 2023 following the lifting of restrictions. There was also an atypical out-of-season prevalence with a peak in March and April 2024. HPIV demonstrated a regular prevalence pattern with annual peaks in November, except for another peak in May 2021. An out-of-season peak was also observed soon after the lifting of restrictions in June 2023, as well as in 2024. The seasonal patterns of HBOV was regular with annual peaks in November, and an out-of-season peak was also observed in June 2023. HMPV and HCOV exhibited irregular prevalence patterns during COVID-19, yet both were active following the lifting of restrictions.

### Co-infections during and post the COVID-19 periods

**Fig. 4:**
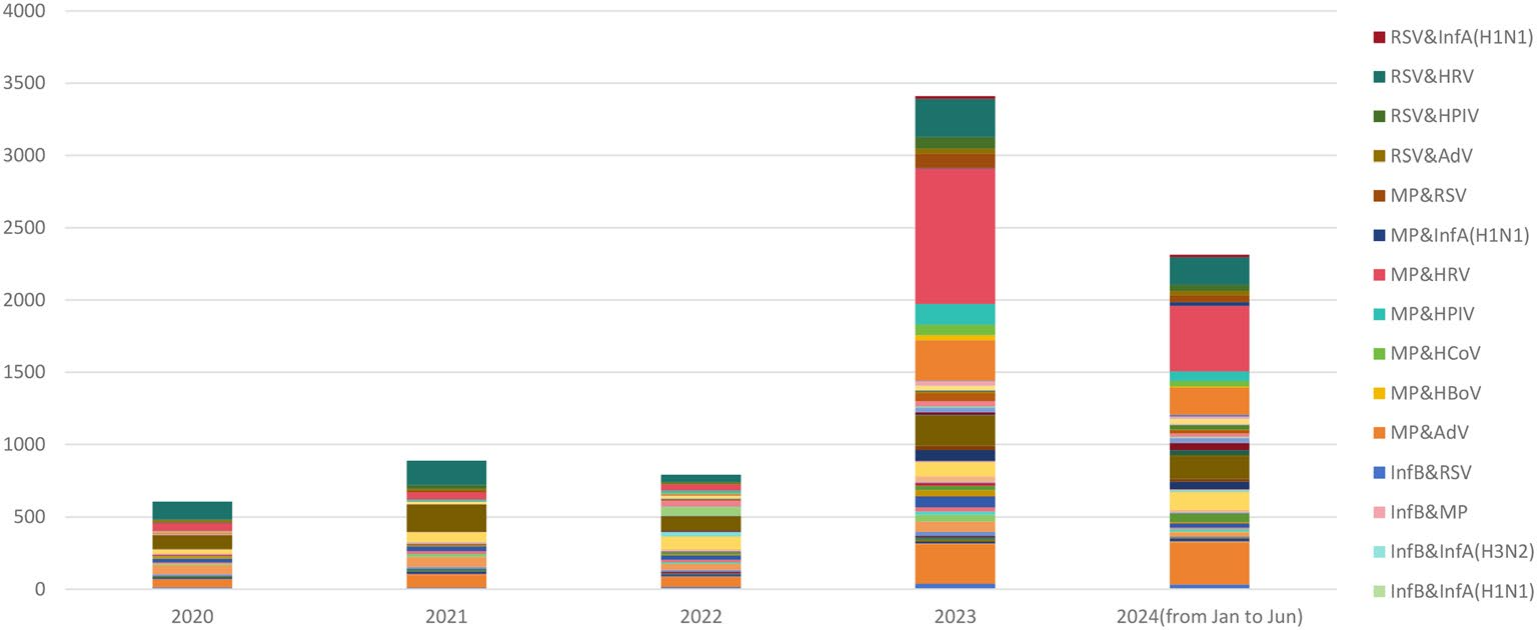
The co-infection pathogens and cases in 2020 to 2024(from Jan to Jun)

The prevalence rate of co-infections was 15.1% in the post-COVID-19 period, significantly higher than the 6.3% during COVID-19 (Table 1). In 2024, the prevalence rate of co-infections was 17.4%, even higher than the 13.9% observed in 2023 (χ²=28.536, *P*<0.001) (Table 1). The patterns and cases of co-infection are illustrated in Fig. 4. The leading five co-infection pathogen patterns are shown in Table 2, and these patterns were identical in both 2023 and 2024. HRV was the most frequently involved pathogen in co-infections, combining with RSV in 2020, with HPIV in 2021 and 2022, and with *MP* in 2023 and 2024 as the leading combinations (Table 2).

**Table 2:** The leading five co-infection pathogens pattern in 2020 to 2024(from Jan to Jun)

Respiratory pathogens	2020	*N*=(7185)	2021	(*N* = 12722)	2022	(*N* = 12824)	2023	(*N* = 20284)	2024(from Jan to Jun)	(*N* = 10999)
	*n*	(%)	*n*	(%)	*n*	(%)	*n*	(%)	*n*	(%)
*MP*&HRV	55	(0.8%)	NA	NA	44	(0.3%)	938	(4.6%)	453	(4.1%)
AdV&HRV	52	(0.7%)	88	(0.7%)	68	(0.5%)	274	(1.4%)	292	(2.7%)
RSV&HRV	122	(1.7%)	165	(1.3%)	49	(0.4%)	264	(1.3%)	191	(1.7%)
HPIV&HRV	92	(1.3%)	188	(1.5%)	98	(0.8%)	209	(1.0%)	156	(1.4%)
*MP*&AdV	NA	NA	NA	NA	NA	NA	281	(1.4%)	189	(1.7%)
HMPV&HRV	NA	NA	66	(0.5%)	90	(0.7%)	NA	NA	NA	NA
HBoV&HRV	65	(0.9%)	71	(0.6%)	NA	NA	NA	NA	NA	NA
Others	156	(2.2%)	223	(1.8%)	358	(2.8%)	862	(4.2%)	629	(5.7%)
All	542	(7.5%)	801	(6.3%)	707	(5.5%)	2828	(13.9%)	1910	(17.4%)

NA: not available

### Prevalence of pathogens by gender and different age groups

**Fig. 5:**
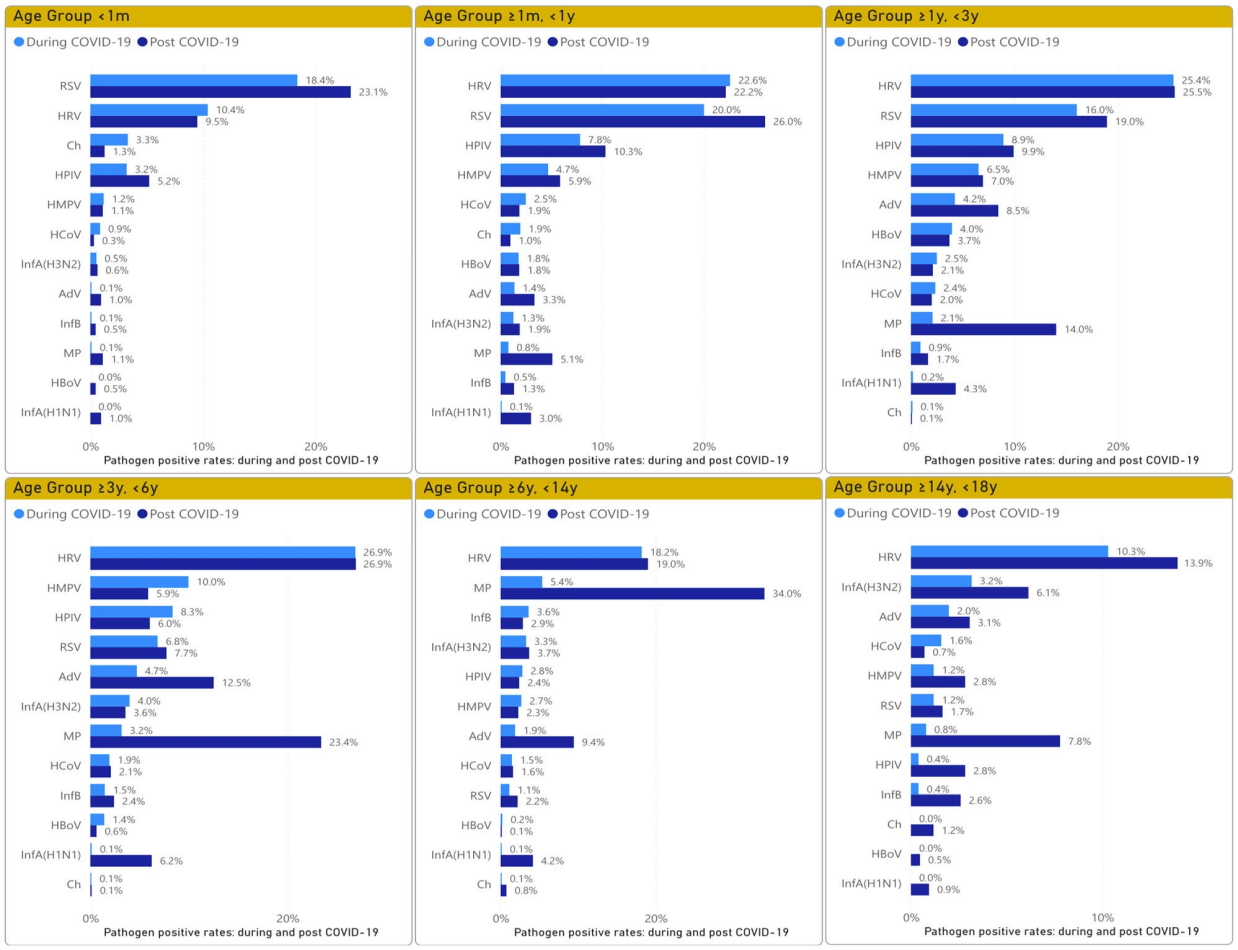
The pathogens detection rates in different age groups during and post the COVID-19 periods

The detection rates in both males and females increased in the post COVID-19 period. Similarly, the detection rates in all six age groups also increased in the post COVID-19 period (Table 3). The prevalence of pathogens in different age groups during and post the COVID-19 periods is illustrated in Fig. 5. HRV was the leading pathogen in all age groups, except in the < 1 m group, where RSV was predominant. In the ≥ 1 m, <1y and ≥ 1y, <3y groups, RSV was in the second place, followed by HPIV and HMPV, with the detection rate of RSV being significantly higher than that of HPIV and HMPV. In the ≥ 3y, <6y group, the leading four viral pathogens were HRV, HMPV, HPIV, and RSV. In ≥ 6y, < 14y, and ≥ 14y, < 18y groups, *MP* and influenza were the main pathogens. In the post COVID-19 period, *MP* increased and became a main pathogen in the ≥ 3y, <6y, ≥6y, < 14y, and ≥ 14y, < 18y groups. *MP* also showed an increase in the ≥ 1 m, <1y and ≥ 1y, <3y groups, becoming a main pathogen in the ≥ 1y, <3y group.

**Table 3:** The detection rates by gender and different age groups during and post the COVID-19 periods

	During COVID-19(from Jan 2020 to Dec 2022)			Post COVID-19(from Jan 2023 to Jun 2024)			χ²	*P*
	Positive cases(*n*)	Overall(*N*)	Positive rate	Positive cases(*n*)	Overall(*N*)	Positive rate		
Gender								
Male	11,456	19,585	58.5%	13,218	18,207	72.6%	828.291	<0.001
Female	7,394	13,164	56.2%	9,314	13,076	71.2%	643.352	<0.001
Age groups								
<1 m	340	930	36.6%	281	642	43.8%	8.262	0.004
≥ 1 m, <1y	5,067	8,692	58.3%	4,689	6,814	68.8%	181.155	<0.001
≥ 1y, <3y	6,206	9,573	64.8%	5,909	7,496	78.8%	400.033	<0.001
≥ 3y, 6y	5,493	8,862	62.0%	7,007	8,971	78.1%	552.883	<0.001
≥ 6y, <14y	1,688	4,431	38.1%	6,314	9,251	68.3%	1122.186	<0.001
≥ 14y, <18y	56	261	21.5%	183	445	41.1%	28.418	<0.001
Respiratory pathogens								
AdV	1,026	32,749	3.1%	2,663	31,283	8.5%	852.869	<0.001
Ch	227	32,749	0.7%	159	31,283	0.5%	9.128	0.003
HBoV	668	32,749	2.0%	438	31,283	1.4%	38.564	<0.001
HCoV	690	32,749	2.1%	572	31,283	1.8%	6.421	0.011
HMPV	2,043	32,749	6.2%	1,551	31,283	5.0%	49.511	<0.001
HPIV	2,432	32,749	7.4%	2,090	31,283	6.7%	13.539	<0.001
HRV	7,704	32,749	23.5%	7,160	31,283	22.9%	3.637	0.057
InfA(H1N1)	41	32,749	0.1%	1,375	31,283	4.4%	1349.100	<0.001
InfA(H3N2)	856	32,749	2.6%	911	31,283	2.9%	5.306	0.021
InfB	418	32,749	1.3%	661	31,283	2.1%	67.592	<0.001
MP	783	32,749	2.4%	6,226	31,283	19.9%	5033.067	<0.001
RSV	4,126	32,749	12.6%	3,947	31,283	12.6%	0.005	0.945

### ICU admission rate in ARI with each pathogen during and post the COVID-19 periods

There was no significant difference in ICU admission rates during and post the COVID-19 periods, except for HBoV (Table 4). The ICU admission rate for HBoV infection was 8.7% in the post COVID-19 period, which was higher than the 4.9% during COVID-19 period. In cases of ICU admissions with *Ch* infection, 17 out of 23 were in the neonatal ICU.

**Table 4:** ICU admission rate of each pathogen during and post the COVID-19 periods

Respiratory pathogens	During COVID-19			Post COVID-19			χ²	*P*
	ICU admission cases	Positive_Total	ICU admission rate	ICU admission cases	Positive_Total	ICU admission rate		
AdV	12	1,026	1.2%	53	2,663	2.0%	2.882	0.09
Ch	13	227	5.7%	10	159	6.3%	0.053	0.818
HBoV	33	668	4.9%	38	438	8.7%	6.145	0.013
HCoV	20	690	2.9%	11	572	1.9%	1.242	0.265
HMPV	31	2,043	1.5%	25	1,551	1.6%	0.051	0.821
HPIV	43	2,432	1.8%	51	2,090	2.4%	2.494	0.114
HRV	203	7,704	2.6%	187	7,160	2.6%	0.008	0.929
InfA(H1N1)	1	41	2.4%	44	1,375	3.2%	0.075	0.784
InfA(H3N2)	28	856	3.3%	35	911	3.8%	0.418	0.518
InfB	6	418	1.4%	19	661	2.9%	2.343	0.126
MP	6	783	0.8%	52	6,226	0.8%	0.040	0.841
RSV	112	4,126	2.7%	121	3,947	3.1%	0.887	0.346

## Discussion

The current study indicates that the COVID-19 pandemic and NPI measures, as well as the lifting of these restrictions, have influenced the epidemiological patterns of multiple pathogens. It corroborates and extends previous findings from our institute [10, 11], demonstrating a significant reduction in ARI admissions and prevalence of pathogens due to the enforcement of lockdown and strict NPI measures in response to the COVID-19 pandemic in early 2020. However, many pathogens, such as influenza, re-emerged irregularly relative to seasonal patterns following the gradual relaxation of these restrictions from 2021 to 2022. Consistent with the warnings proposed in previous studies [4], several months after the cessation of the “zero-COVID-19” policy and restrictions in early 2023, almost all pathogens, except *Ch*, re-emerged and contributed to a marked increase in ARI hospitalizations, comparable to the pre-pandemic era as documented in our previous work [11]. The marked increase in co-infection rates and the significant rise in pathogen detection rates across all age groups suggest a concurrent prevalence of multiple pathogens in the post-pandemic period. This may be associated with the reduction in pathogen exposure due to various NPI measures during the pandemic, leading to a decrease in protective antibodies and increased susceptibility upon re-exposure to pathogens [12]. The prevalence of *MP* and ADV remained consistent throughout the pandemic, while the seasonal epidemic peak of RSV disappeared in the last year of the pandemic; however, all of them experienced an explosive epidemic in the first year of post-pandemic. Furthermore, the epidemiological peak of *MP* and ADV were higher than the peaks observed during the pre-pandemic seasons [11]. The resurgence of respiratory pathogens was noted extensively in various districts of China [9, 13, 14] and globally after the lifting of restrictions [8, 15–18]. Notably, in the current study, the high levels of ARI hospitalization and pathogen detection rates continued into the second year of the post-COVID-19 period in 2024, suggesting a lasting impact on the prevalence patterns of pathogens. This underscores the importance of available vaccinations and appropriate NPI measures.

RSV is an important pathogen causing respiratory infections in children. In the current study, RSV was the second most common pathogen, closely following HRV, in the 1 month to 3-year-old age group. A decline of RSV during the pandemic [19], and a significant resurgence, including atypical out-of-season outbreaks, has been documented in Europe [7, 20, 21], Australia [18], Asia [22], and the United States [23, 24] following the relaxation of NPI measures. In Shenzhen, the typical season of the RSV epidemic was in the summer, as observed in this study in 2020 and 2021. However, in 2022, there was no typical seasonal RSV prevalence. An atypical seasonal epidemic occurred in March-April 2023 following the lifting of restrictions. The same pattern was documented in Zhejiang and Jiangshu province of China [12, 25], where the RSV seasonality and transmission zones were different from those in Shenzhen [26]. It is worth noting that another atypical seasonal epidemic re-emerged in February-April 2024 in the current study, indicating that the disruption of RSV prevalence patterns by the pandemic and NPI measures is not a one-time event. While the first wave of RSV epidemics following pandemic suppression exhibited unusual patterns, the second and third waves more closely resembled typical RSV patterns in many countries [27]. The onset and peak timings of future epidemics following the disruption of normal RSV dynamics need close monitoring to inform the delivery of preventive and control measures.

*MP* is recognized as a significant pathogen among children with CAP, particularly prevalent in those aged five years and older [1]. *MP* infections are known to be endemic, exhibiting periodic epidemic peaks at irregular intervals. Throughout the COVID-19 lockdown period, epidemiological surveillance consistently reported low levels of *MP* [11, 28]. Our findings indicate an outbreak of *MP* commencing in May 2023, a few months after the lifting of restrictions, continuing into 2024, not only among susceptible children over 6 years old but also with increased detection rates in children under 6 years old. A similar pattern was observed in Shanghai, Eastern China, with a peak in *MP* infections noted since June 2023 [29]. The extensive epidemic of *MP* across China has garnered significant concern [30, 31]. Nevertheless, despite the easing or discontinuation of NPI measures, a sustained decrease in *MP* incidence was documented across 20 countries spanning Europe, Asia, the Americas, and Oceania [32], persisting until at least September 2023 [33]. The occurrence of an *MP* outbreak in China, contrasted with a delayed resurgence in other countries, suggests a pathogen-specific and regionally nuanced epidemiological pattern disruption due to the pandemic.

Influenza virus prevalence was nearly eradicated during 2020 and 2021 [11], a trend consistent with findings from the United States [15] and Western Australia [34]. However, our ongoing surveillance data indicate a resurgence of InfB in the winter of 2021 and an outbreak of InfA(H3N2) from April to July 2022. A resurgence of InfA(H1N1) was observed until after the lifting of restrictions, with an outbreak occurring from February to May 2023. The epidemiological peak of InfA was higher than the peaks observed during the pre-pandemic seasons [11]. These observations suggest that the pandemic and NPI measures have disrupted the traditional seasonal circulation patterns of influenza. It is crucial to enhance epidemiological surveillance for influenza to avert potential public health crises. Notably, in the winter of 2023 and spring of 2024, InfA(H3N2), InfA(H1N1), and InfB all re-emerged.

HRV is the most common viral pathogen across all age groups except for newborns in this study, exhibiting a stable epidemiological pattern and not appearing to be affected by the pandemic and NPI measures. The detection rate of HRV remained high during the COVID-19 period, consistent with other studies [15, 35–37], which might be due to the ineffectiveness of alcohol-based hand sanitizers and the weakened inhibitory effect of face masks against rhinovirus [38, 39]. Consistent with this, Xu et al. [40] found that in 2020, the positive rates of Mp, ADV, HRV, PIV, and influenza significantly decreased compared to the pre-pandemic period. Meanwhile, HRV was the most commonly detected respiratory pathogen and the most frequent across all five age groups. Following household exposure, children were significantly less likely to become infected with SARS-CoV-2 compared to HRV (aOR 0.16), whereas this trend was opposite in adults (aOR 1.71) [41]. The epidemiological patterns of HPIV, HMPV, and HCOV were also minimally affected during the pandemic. HPIV and HMPV are common respiratory pathogens that typically follow RSV in prevalence in children aged 3 to 6 years, with the positivity rates for both being similar to those of RSV.

The discontinuation of NPI measures increased the opportunity for pathogen infection; whether this also results in an increase in the severity remains a significant concern. In the current study, there was no significant difference in ICU admission rates for all pathogens except HBOV during and after the pandemic. The ICU admission rate of HBOV infection was 4.9% during the pandemic, close to 4.2% in our previous study with data up to January 2022 [11]. Notably, in the post-pandemic period, it increased to 8.7%. HBOV was uncommon in our study, with the highest detection rate of 4.0% in children 1–3 years old. However, studies have indicated that HBOV can cause severe ARI in children in the absence of viral and bacterial co-infections [42]. At Children’s Hospital of Soochow University in China, for lower respiratory tract infections by HBOV from January 2017 to October 2021, the rate of admission to ICU was 9.6%, which was much higher than that in the hMPV group (1%) [43]. The rate of HBOV associated admission to ICU was 7.3% [44] and 11.6% [45] in two children’s hospitals in Italy. The mechanism of severe ARI associated with HBOV and its worsening in the post-pandemic period is yet to be elucidated. This highlights the importance of persistent epidemiological surveillance of HBOV. The ICU admission rate of *Ch* seemed high in both the during and post-pandemic periods, though there was no difference between these two periods. It should be noted that *Ch* was a main pathogen in children less than one month old, and rare in other age groups, with most of the ICU admission cases being hospitalized in the neonatal ICU.

The limitations of this study include that all data were sourced from a single hospital and only included hospitalized ARI cases, which may introduce selection bias. Additionally, the study did not account for trends in bacterial pathogens. The current data indicate that the implementation of NPI measures in response to the COVID-19 pandemic has significantly reshaped the epidemiological patterns, even the clinical manifestations of some respiratory pathogens. As restrictions were lifted, continuous monitoring of these patterns is essential to understand the duration of these effects and to inform effective response strategies.

## Data Availability

Data is provided within the manuscript or supplementary information files. The dataset generated and analyzed during the current study is not publicly available as it contains protected health information, but the de-identified dataset is available from the corresponding author on reasonable request.

## Abbreviations

COVID-19: Coronavirus Disease 2019
ARI: Acute respiratory infections
HBOV: Human Bocavirus
MP: Mycoplasma pneumoniae
ADV: Adenovirus
RSV: Respiratory Syncytial Virus
CAP: Community-acquired pneumonia
NPI: Non-pharmaceutical interventions
PCR: Polymerase chain reaction
HPIV: Human parainfluenza virus
HMPV: Human metapneumovirus
HRV: Human rhinovirus
HCOV: Human coronavirus
Ch: Chlamydia
ICU: Intensive Care Unit
